# Polyphenols, the Healthy Brand of Olive Oil: Insights and Perspectives

**DOI:** 10.3390/nu13113831

**Published:** 2021-10-27

**Authors:** Mauro Finicelli, Tiziana Squillaro, Umberto Galderisi, Gianfranco Peluso

**Affiliations:** 1 Research Institute on Terrestrial Ecosystems (IRET), National Research Council of Italy (CNR), Via Pietro Castellino 111, 80131 Naples, Italy; 2 Department of Experimental Medicine, University of Campania “Luigi Vanvitelli”, Via Santa Maria di Costantinopoli 16, 80138 Naples, Italy; tiziana.squillaro@unicampania.it (T.S.); umberto.galderisi@unicampania.it (U.G.)

**Keywords:** olive oil, polyphenols, clinical trial, metabolism, human health, bioavailability

## Abstract

Given their beneficial potential on human health, plant food bioactive molecules are important components influencing nutrition. Polyphenols have been widely acknowledged for their potentially protective role against several complex diseases. In particular, the polyphenols of olive oil (OOPs) emerge as the key components of many healthy diets and have been widely studied for their beneficial properties. The qualitative and quantitative profile defining the composition of olive oil phenolic molecules as well as their absorbance and metabolism once ingested are key aspects that need to be considered to fully understand the health potential of these molecules. In this review, we provide an overview of the key aspects influencing these variations by focusing on the factors influencing the biosynthesis of OOPs and the findings about their absorption and metabolism. Despite the encouraging evidence, the health potential of OOPs is still debated due to limitations in current studies. Clinical trials are necessary to fully understand and validate the beneficial effects of olive oil and OOPs on human health. We provide an update of the clinical trials based on olive oil and/or OOPs that aim to understand their beneficial effects. Tailored studies are needed to standardize the polyphenolic distribution and understand the variables associated with phenol-enriched OO. An in-depth knowledge of the steps that occur following polyphenol ingestion may reveal useful insights to be used in clinical settings for the prevention and treatment of many diseases.

## 1. Introduction

**Figure 1:**
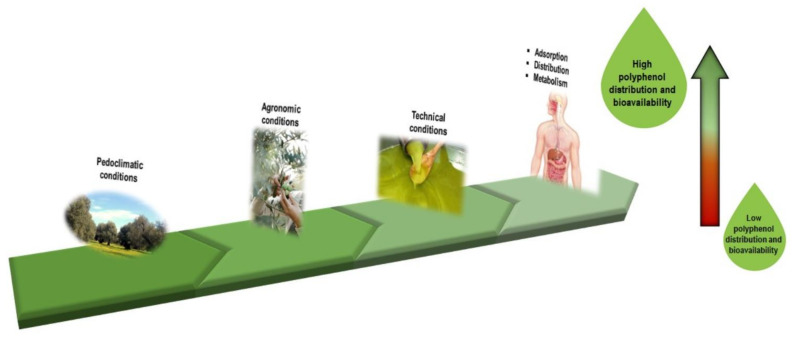
A representative illustration of the variations influencing polyphenol distribution and bioavailability.

Nutrition is fundamental for the correct sustainment of the body as well as for the maintenance of optimal health [1]. Modern nutrition is a multidisciplinary science encompassing evidence from epidemiology, biochemistry, behavioral science, biology, food science, and medicine [2]. Without these understandings, the real power of foods will remain unknown to scientists, and nutrition will be a black box with no scope for improvement [2,3]. In addition to macro- (carbohydrate, fat, and protein) and micro-nutrients (vitamins and minerals), other molecules daily consumed in foods exert their potential for protecting cells and tissues from stress and helping to improve long-term well-being [2]. Plant food bioactive molecules are important components of the human diet and have beneficial effects on human health, although they have not been categorized as essential nutrients. Among them, polyphenols have received considerable attention from the scientific community for their potentially protective role against several complex diseases [4]. In particular, the polyphenols of olive oil (OOPs) have been widely studied for their beneficial properties on human health and metabolism; they have also been studied because of olive oil’s popularity in many healthy diets [5,6]. The potential health benefit of dietary OOPs depends upon many factors. The qualitative and quantitative profile defining olive oil phenolic composition has to be considered. This refers to the modifications in polyphenol content due to agronomic, pedoclimatic, and technological conditions [7,8] (Figure 1). Other important features composing these variations are the differences in adsorption, distribution, and metabolism of the polyphenols (Figure 1). Indeed, once ingested, OOPs give rise to different metabolites, which, along with parent compounds, are able to reach tissue concentrations and exert beneficial effects [8].

In this review, we provide an overview of these aspects, focusing on the endogenous and exogenous factors influencing the biosynthesis of OOPs and the findings about their absorption and metabolism. Moreover, we give an update on clinical trials based on olive oil and/or OOPs. This is informative about the existing studies aiming to understand the beneficial effects of olive oil (OO) and/or its polyphenols.

## 2. Olive Oil Polyphenols

*Olea europea* L. is a well-known evergreen tree native to the Mediterranean basin and characterized by a slow-growing rate and an extremely long life expectancy of up to 1000 years [9,10]. This species is one of the most important trees for the Mediterranean economy, providing many commercial products such as food, lumber, and cosmetics. Nevertheless, the most important product supplied by *Olea eueopea* L. is OO [9,11]. Indeed, the beneficial effects of the Mediterranean diet (MD) are globally acknowledged. OO, especially extra-virgin olive oil (EVOO), is now recognized as a symbol of the MD. The high consumption of EVOO, ranging from 15.3 to 23 kg per capita/year [12], is a staple of the MD and is one of the major differences with other healthy diets [11,12]. The positive health effects of a MD rich in EVOO have been demonstrated for Type 2 diabetes and cancer as well as neurodegenerative and cardiovascular diseases [5,11,13,14,15,16,17]. Much evidence describes the beneficial impact of EVOO as the result of its specific components. The high content of monosaturated fatty acids (C18:1, ranging from 55 to 83% of the total fatty acids) has been widely associated with the nutritional and health-promoting properties of this food [7,11]. Interestingly, the attention of researchers has mainly focused on the lesser component of olive oil (2% of total weight), which is rich in bioactive molecules [5].

Among them, phenolic compounds are characterized by a broad spectrum of biological activities ranging from auto-oxidation stability to the beneficial effects on human health [18]. Given their well-established activities, OOPs have demonstrated their effect in preserving the stability and organoleptic properties of OO [19]. Nevertheless, OOPs are extensively studied for their health-promoting properties because of the widely acknowledged antioxidant, anti-inflammatory, cardioprotective, neuroprotective, anticancer, antidiabetic, and antimicrobial properties [7,20,21,22,23].

**Figure 2:**
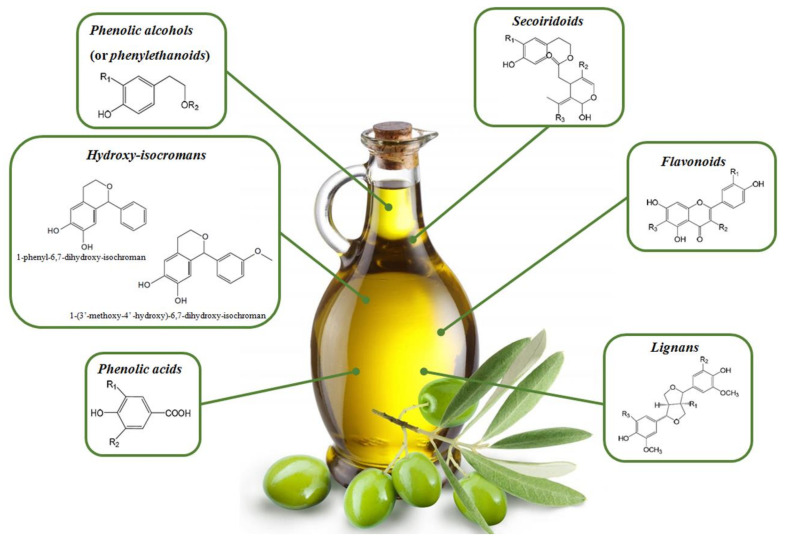
A representative scheme evidencing the principal classes of polyphenols in olive oil.

These molecules belong to the hydrophilic phenolic fraction constituting EVOO and are present in free, bound, or esterified forms [24]. More than 30 different OOPs were identified in EVOOs with a total phenolic range varying between 50 and 800 mg/kg [8,25,26,27]. According to their chemical structure, OOPs are categorized as follows (Figure 2):

- Secoiridoids are phenolic compounds found in abundance in O. europea with respect to other plant species. They are chemically characterized by a phenyl ethyl alcohol (3,4-DHPEA or p-HPEA) linked to elenolic acid or its derivates; in most cases, they are glycosylated [7]. Secoiridoids are one of the most important micronutrients in EVOO [24]. Demethyloleuropein, oleuropein (Ole), and ligstroside (Lig) are the main glycosides present in olive fruit and their aglycones, accounting for 90% of the phenolic compounds in EVOO [28]. Interestingly, the bitter taste of OO is the result of the secoiridoid content, especially the dialdehydic form of Ole aglycone [29].
- Phenolic alcohols (or phenylethanoids) possess a hydroxyl group attached to an aromatic hydrocarbon group. The main molecules encompassed in this class are hydroxytyrosol (3,4-dihydroxyphenyl ethanol or 3,4 DHPEA; HTyr), tyrosol (p-Hydroxyphenyl ethanol or p-HPEA; Tyr), and oleocanthal [8]. Htyr and Tyr are present in low concentrations in fresh OO, but their amount increases substantially during the storage process because of the hydrolysis of secoiridoids [30].
- Flavonoids have a chemical structure composed of two benzene rings joined by three linear carbon chains. These molecules undergo further modifications, such as glycosylation, giving rise to other compounds divided in other groups, i.e., flavones, flavonols, flavanones, and flavanols [8]. The first flavonoids identified in virgin OO were flavones; their freeform, luteolin and apigenin, are the most concentrated compounds [31].
- Lignans are chemically characterized by the condensation of aromatic aldehydes. The pulp of olives as well as the woody portion of the seed contain lignans; these molecules are released into the oil during the extraction process without biochemical modifications [31]. (+)-pinoresinol and (+)-1-Acetoxypinoresinol are the lignans most concentrated in EVOO [24].
- Phenolic acids are further subdivided into two groups of hydroxybenzoic acid derivatives (e.g., p-hydroxybenzoic, protocatechuic, vanillic, syringic, and gallic acid) and hydroxycinnamic acid derivatives (e.g., p-coumaric, ferulic, cinnamic caffeic, and synaptic acid [7].
- Hydroxy-isocromans consists of the only two molecules characterized in commercial virgin OO, i.e., 1-phenyl-6,7-dihydroxy-isochroman and 1-(3′-methoxy-4′ -hydroxy)-6,7-dihydroxy-isochroman. These compounds are formed from the HTyr reaction with benzaldehyde and vanillin, respectively [25].

It has been widely acknowledged that HTyr, Tyr, and Ole, the most copious polyphenols in OO, are outstanding for their bioactive features [31]. Interestingly, in May 2012, the European Food Safety Agency (EFSA) [32,33] authorized the claim that “olive oil polyphenols contribute to the protection of blood lipids from oxidative stress.” This claim was valid for those OOs containing at least 5 mg of HTyr and its derivates (e.g., Tyr and Ole) per 20 g of OO.

According to this evidence, in the next paragraphs, we focus our attention mainly on these particular polyphenols.

## 3. The Endogenous and Exogenous Factors Influencing the Biosynthesis of OOPs

The plants produce phenols as secondary metabolites, which are widely distributed through organs to exert their metabolic and physiologic functions, such as maintenance of plant integrity, floral pigmentation, and defense against photogenes [34]. This also occurs in *Olea europea* L., and the amount and distribution of these molecules, especially in olive fruit, is broadly variable, depending on many determinants, such as biochemical, agronomic (i.e., genetics, cultivar, ripening stage, biotic, and abiotic stress), and technological factors [7,35]. The synthesis of OOPs occurs in the olive fruit by the action of chemical and enzymatic reactions induced by endogenous enzymes, such as β-glycosidase, which hydrolyzes phenolic glycosides, and oxidoreductase (e.g., polyphenoloxidase) and peroxidase, which oxidize phenolic compounds [11,36].

The phenolic glycosides, initially present in olive tissue, and the activity of the above-mentioned enzymes are also influenced by agronomic factors, e.g., fruit ripeness. Ole and Lig are the main phenolic glycosides initially present in olive tissue and have correlated to the early-harvest olive because of the high level of β-glycosidase in the green stage [11,37,38,39]. As the ripeness continues (i.e., black stage), the glucosidase activity decreases along with a concomitant decline in phenolic glycoside concentration (Lozano-Castellón et al., 2020). Thus, according to fruit ripeness, the polyphenolic content in OO may vary; some compounds could be not found, or they were present at a very low level [7].

The mechanical techniques used for obtaining OO (mainly crushing and malaxation) also impact the release and activity of the endogenous enzymes of the olive fruits. Indeed, enzymatic activity can be modulated by controlling malaxation duration and atmospheric conditions inside the malaxer. So, the concentration of Ole increases as the temperature of the olive paste rises (up to 30 and 35 °C) during malaxation [11,38,40].

In addition to these factors, the composition and concentration of polyphenols in OO and EVOO is affected by pedoclimatic conditions. Indeed, soil characteristics, precipitation, temperature, and humidity may determine the phenolic chemical profile in plants and thus in OO [7,34]. Bakhouche et al. analyzed the phenolic content of Arbequina EVOOs obtained by olives cultivated in different locations in southern Catalonia (Spain). Thirty-two OO samples were analyzed, and quantitative differences in phenolic compounds were evidenced. Accordingly, the authors concluded that the phenolic content of EVOO seemed to depend highly on geographical area [41]. An interesting study analyzed the phenolic profile of EVOO derived from the Maltese islands. The authors isolated and analyzed the polar fractions of EVOOs obtained from nine indigenous cultivars, 12 foreign but locally grown cultivars, and 32 foreign EVOOs. Their data showed differences in locally grown cultivars compared to the same cultivar grown in another country. This has supported the findings that certain polyphenols in olive trees are dependent on pedoclimatic conditions and not solely on genetic factors [42].

Finally, during OO storage, phenolic compounds undergo quantitative and qualitative modifications because of the occurrence of oxidative and hydrolytic reactions. For instance, the level of simple phenols, such as HTyr, increases during the storage of OO because of the hydrolysis of complex polyphenols (e.g., secoiridoids) [18,43]. Kotsiou and Tasioula-Magari analyzed the quantitative variation of phenolic compounds of five EVOO samples belonging to five Greek olive varieties stored in dark glass bottles without central heating for 24 months. Their data showed a decrease in secoiridoid derivatives with a concomitant increase in simple polyphenols, i.e., HT and Tyr, because of the storage-induced hydrolytic and oxidative effects [43].

All these findings demonstrate the high number of variables influencing the quantitative and qualitative polyphenolic profile of OO. This suggests the need for standardized procedures to maximize the healthy properties of OO.

## 4. Absorption and Metabolism of OOPs

The average value of OO intake in the MD is estimated to be around 25–50 mL per day [44]. This is associable with a consumption of 9 mg of OOPs, of which 1 mg consists in HTyr and Tyr and the other 8 mg encompasses their elenolic esters and Ole- and Lig-aglycole [8,44]. The promising beneficial effects of OOPs are strongly influenced by the degree to which these molecules are bioavailable: namely, whether the active compound is adsorbed and metabolized, becoming available in the site of action in specific tissue or organs [7,8]. Thus, once in the body, the bioaccessibility and bioavailability (depending on the absorption, colonic fermentation, and metabolism of these molecules) are other key steps driving the variations in polyphenolic distribution [7]. The absorption and metabolism of phenolic compounds are complex and not fully understood. They are influenced by several factors such as physiochemical characteristics, basic structural properties, polarity, degree of polymerization or glycosylation, and solubility [11,45,46,47,48]. The metabolism of OOPs (e.g., due to microbiota activity) can induce modifications leading to molecules differing from the parental one, bestowed with different biological activities and bioavailability. For instance, a difference in the metabolism of HTyr exists among the free form and its natural precursors, such as Ole or aglycone forms such as secoiridoids. Lopez de la Hazas et al. showed that diet supplemented with Ole induced the maximal bioavailability oh HTyr. They hypothesized that the higher stability of Ole during digestive process may be accountable for its major exposure in phase II metabolism [46].

OOPs undergo phase I (mainly hydrolysis) and phase II (involving methylation, sulfation, and glucuronidation) metabolism, which take place essentially in the stomach, enterocytes, and liver; they are also modified by the action of gut microbiota [25,49].

### 4.1. Absorption

After ingestion, OO produces a micellar solution, resulting in an aqueous and lipid phase. A first modification takes place in the mouth because of the hydrolytic action of saliva. Then, OOPs reach the stomach where they are partially modified (hydrolyzed) before passing into the small intestine. Aglycole-secoiridoids are susceptible to a gastric environment where they are hydrolyzed, resulting in a significant increase in their derivates, i.e., free HTyr and Tyr. This process is time-dependent, increasing as residence in the stomach continues. Nevertheless, some of them remain unhydrolyzed under normal pH conditions and physiological time frames (pH 2.0 and up to 4 h, respectively). Glycosylated secoiridoids (e.g., Ole), are also not susceptible to a gastric environment [8,50,51]. Therefore, these molecules reach the small intestine unmodified and with a large amount of HTyr and Tyr. The latter are the best absorbed phenolics in the intestine tract (absorption rate 40–95%), which allow the achievement of a peak concentration in human plasma 1 h following ingestion [7,47].

A widely acknowledged mechanism, accounting for the absorption of HTyr and Tyr, is the passive bidirectional transport occurring through the membrane of human enterocytes [52,53]. Further studies have evidenced the matrix-dependent absorbance of these molecules. The oil matrix seems to enhance the intake of these phenols with respect to water solutions or yogurt [54,55].

The larger phenolic compounds undergo a different absorption route. Various mechanisms have been proposed for Ole-glycoside, such as a glucose transponder, paracellular way, or transcellular passive diffusion [52,56].

All phenolic molecules that are not absorbed in the small intestine pass through the colon where they can be fermented by gut microbiota. Studying the interaction between OOPs and bacteria is important to understanding the beneficial potential of these molecules. The presence of polyphenols in colonic environment may play a dual role. First, colon bacteria favor the degradation of some unabsorbed phenolic compounds, providing a wide range of metabolites, which may be adsorbed or excreted. Mosele et al. used an in vitro model by means of human fecal microbiota to study the colonic metabolism of the main OOPs. Their data evidenced a high degradation of HTyr-acetate and Ole in the fecal culture medium [57]. This is of interest, given that Ole reaches the large intestine as an unmodified compound. These data corroborate previous evidence reporting the human colonic bacteria could catabolize Ole into HTyr [50].

On the other hand, unmodified polyphenols and/or their metabolites arriving in the large intestine could also exert a beneficial role by promoting intestinal homeostasis as well as exerting a prebiotic-like effect, influencing the microbiota composition and inhibiting the growth of harmful bacteria [52]. Several studies reported that both non-absorbable phenols or those before absorption seemed to protect the intestinal mucosa from the harmful effect of oxidized species at the colon level. This effect could result in antagonizing the action of unsaturated fatty acids and oxidized cholesterol products consumed with a normal diet [7,58]. Santos et al. studied the in vitro ability of six bacterial strains in converting Ole into HTyr. Their results showed that microorganisms belonging to the genera Lactobacillus, Bifidobacteria, and Enterococcus were able to catalyze this hydrolysis [59]. Given that Ole seemed to be a carbon source for Lactobacillus and Bifidobacteria but not for other strains, such as Clostridium and E. Coli, this molecule could have a prebiotic potential. Accordingly, it has been hypothesized that OOPs play a role in influencing microbiota composition, disadvantaging pathogenic bacteria. HTyr showed a significant antimicrobial activity once exposed to selected Enterobacters species [60]. Ole was able to delay the growth and toxin production of Staphylococcus aureus at low concentrations (0.2% *w*/*v*) as well as to exert an inhibitory effect for Mycoplasma species [61,62,63].

Although this evidence reveals the role of OOPs in modulating microbiota and the ability of gut microbiota to metabolize OOPs, these aspects need to be further investigated to disclose therapeutic potentialities [64].

### 4.2. Metabolism

Once absorbed, OOPs must be distributed and metabolized. To better address this evidence, we focus on the metabolites of the widely and abundantly distributed OOPs, such as HTyr, Tyr, Ole, Oleochantal (Oc), and Lig [7,20].

#### 4.2.1. HTyr

HTyr and its metabolites show a widespread distribution, with a prevalence in muscle, testis, liver, kidney, and brain [20,65,66]. Of note, HTyr passes across the blood–brain barrier and seems to be a dopaminergic neuronal protector [67,68]

HTyr phase I metabolism takes place by the action of cytosolic non-microsomal alcohol and aldehyde dehydrogenases (ADH and ALDH, respectively) [69]. The latter enzyme oxidizes 3,4-dihydroxyphenylacetaldehyde (DOPAL), which is an important metabolite of the major brain neurotransmitter dopamine. DOPAL is unstable and toxic, so ALDH catalyzes its conversion in 3,4-dihydroxyphenylacetic acid (DOPAC), which can be in turn transformed in HTyr by DOPAC reductase [20,46,66].

Sulfotransferases (SULT), uridine 5′-diphosphoglucuronosyl transferases (UGT), catechol-O-methyltransferase (COMT), and acethyltransferases are the enzymes involved in HTyr phase-II metabolism [20,69]. They give rise to the main metabolites of HTyr described so far, such as O-methylated forms, sulfates, glucuronides, and acetylated and sulfated derivates [69,70,71,72].

The O-methylated forms catalyzed by COMT are important HTyr metabolites. Among them, 3-hydroxy-4-methoxyphenylethanol (homovanillyl alcohol-HVAlc) and 4-hydroxy-3-methoxyphenylacetic acid (homovanillic acid-HVA) result from the action of COMT on HTyr and DOPAC, respectively [72,73].

HTyr can also be a substrate for acetyltransferases, which give rise to HTyr1-acetate by transferring an acetyl group from acetyl-CoA [20]. This metabolite can be further processed by SULT, giving rise to HTyr1-acetate-4′-O-sulfate [20,46]. It should be noted that this molecule, along with HTyr sulfate, are the main metabolites detected in human plasma upon normal dietary consumption of HTyr [46,66].

Sulfated and glucuronidated HTyr are the main metabolites detected in human plasma and urine [8]. Interestingly, studies on rats evidenced a dose-dependent variation of these forms. At a lower dose of HTyr administration (1 mg/kg), the glucuronidation pathway seems to be prevalent to sulforation (25–30% vs. 14%, respectively). As the dose increases (100 mg/kg), this ratio changes, and sulfation is the dominant pathway (75%) [69,74].

As stated before, another way by which the biotransformation of HTyr takes place is through the action of gut microbiota. These microorganisms catabolize the unmetabolized native OOPs by means of oxidation and dehydroxylation reactions. The primary metabolized forms of HTyr are phenylacetic acid (PA) and its derivates; also, phenylpropionic (PP) derivates are the primary product of hydroxytyrosol acetate catabolism [18,20].

Finally, it should be noted that HTyr and its metabolites are excreted mainly by the kidney, and the time required to complete their elimination from the human body is 6 h [18,69]. D’Angelo et al. evidenced a nephroprotective action of HTyr in rats because it remains in the kidney until excretion [66]. Moreover, the recycling route by which HTyr metabolites pass back from the liver to the duodenum via biliary ducts is another mechanism accounting for the longer presence of these molecules in the body [20,46].

#### 4.2.2. Tyr

Tyr is bioavailable in humans even from moderate OO consumption [20]; its half-life in humans ranges from 2 to 4 h [75]. This molecule undergoes an extensive metabolism, so the concentration of its metabolites in biological fluids is much higher than in the free form [76].

Similarly to HTyr, Tyr is also endogenously formed by the oxidative metabolism of tyramine, a monoamine compound resulting from the decarboxylation of tyrosine. Tyramine is the substrate of monoamine oxidase, generating an aldehyde intermediate that could be either oxidized by ALDH or reduced by ADH. The latter enzymatic reaction generates Tyr, and it is enhanced when associated with alcohol consumption [69]. The first evidence was documented in rats and further confirmed in humans [77,78,79]. Perez-Mana measured the Tyr urinary excretion in healthy volunteers following ethanol intake. Their data showed that Tyr excretion following vodka intake was two-fold higher than detected upon placebo administration [78].

Concerning phase II metabolism, Tyr undergoes preferentially glucuronidation and sulfation, and the resulting principal metabolites are 4′-O-glucuronide and 4′-O-sulfate. The latter compound seemed to exert a beneficial role in liver tissue, confirming the evidence describing sulfation as the leading pathway of Tyr into this organ. It must also be noted that in liver microsomes, Tyr could be interconverted in HTyr by the action of cytochrome P450 (CYP). Further studies, using selective enzymatic inhibitors seemed to identify CYP2A6 and CYP2D6 as the isoforms involved in this reaction [46,69,80].

Finally, the non-digested Tyr is catabolized by gut microbiota, giving rise to the same intermediates described for Htyr [20].

#### 4.2.3. Ole

Despite HTyr and Tyr, less is known concerning the metabolism of Ole for EVOO in humans. This may be due to the fact that the bioavailability of these compounds is influenced by different factors both biological (e.g., gender, genotype, age, interaction with food) and technical (e.g., route of administration, extraction processes, and analytical) methods [81,82]. As discussed before for secoiridoids, Ole resists the acidic conditions of the stomach, remaining stable 2–4 h following incubation in gastric juices [83]. Thus, the pH of the milieu and time of permanence in the stomach profoundly impacts the formation and distribution of Ole metabolites [84]. According to Ole chemistry, Carrera Gonzales et al. proposed that the acidic environment could induce the hydrolysis of β-glycosidic bounds with the release of Ole-aglycone and glucose. The former molecule could be further hydrolyzed into HTyr and elenolic acid. It has been estimated that acidic hydrolysis gives rise to 33% of HTyr from the original amount of Ole [20,84].

de Bock et al. demonstrated that Ole is rapidly adsorbed in the intestine (55–60%) [85], where it can be the substrate for lipases that catalyze the release of HTyr and oleoside [84]. Of note, in the large intestine, Ole is also degraded by microflora, giving rise to HTyr. An interesting in vitro study using human fecal microbiota demonstrated a rapid deglycosylation of Ole (6 h incubation). The resulting Ole-aglycone was the substrate for microbial esterase, which supported the production of HTyr and elenoic acid [57]. Other evidence revealed that lactic acid bacteria (in particular, Lactobacillus plantarum) fostered Ole metabolism in favor of HTyr production [59,86]. It should be noted that the catabolism of Ole gives rise to molecules belonging to the PA and PP families [20].

#### 4.2.4. Others

While studies have given great attention to HTyr, Tyr, and Ole, researchers have scarcely studied the bioavailability and pharmacokinetics of other molecules such as Oc, Olacein, and Lig [11,20].

Nevertheless, evidence reports that the acidic gastric environment leads to a time-dependent hydrolysis of phenolic compounds, such as Oc, Lig, and its aglycones, which, in turn, yield a three-fold increase in free Tyr [50].

Pioneering studies have evidenced that most of the Oc metabolites found in plasma and urine are formed in the small intestine and liver. These metabolites result mainly from hydrogenation, hydration, and hydroxylation (phase I metabolism) reactions [11,87,88]. Of note, some hydrogenate Oc metabolites are further subject to glucuronidation (phase II metabolism) [20,88].

On the basis of the evidence described in this paragraph, the biological effect induced by OOPs needs to be considered according to the possible metabolites generated from the parent molecules. This provides useful insights to better define the real effect induced by the presence and the amount of a particular polyphenol according to the principal metabolites it generates once in the body

## 5. OOPs and Clinical Trials

The potentially beneficial effects of OOPs on human health have been described in many in vivo and in vitro studies, highlighting the antioxidant activity of these molecules as the key aspect of their biological activities. Promising findings report associations between OOPs and the prevention or reduced risk of diseases where oxidative stress and inflammation have a high impact such as cancer, metabolic syndrome, digestive disorders, and cardiovascular diseases [7]. Despite this encouraging evidence, the preventive potential of OOPs is still debated due to limitations in current studies. Thus, clinical trials are necessary to fully understand and validate the beneficial effects of OO and OOPs on human health.

In this section, we report a list of the clinical trials based on OO and/or OOPs searched within the public web archive of ClinicalTrials.gov (15 March 2021) [89], which was updated on 15 March 2021. This resource, provided by the U.S. National Library of Medicine, is a database of privately and publicly funded clinical studies conducted around the world. We conducted multiple searches using the following keywords: Olive Oil Polyphenols, Hydroxityrosol, Hydroxytyrosol Olive Oil, Tyrosol, Tyrosol Olive Oil, Oleuropein, Oleuropein Olive Oil, Oleocanthal, Oleocanthal Olive Oil, Ligstroside, and Ligstroside Olive Oil. These searches allowed us to identify clinical trials with olive oil and/or its polyphenols that suggested either their potential benefits on human health or intriguing clinical implications.

Briefly, the studies resulting from each single search were compared with the others; duplicates were eliminated. This allowed us to identify 48 studies. Of these, 11 were eliminated because of their design (i.e., NCT03921580, NCT03482401, NCT04783714, NCT04756310, NCT01381354, NCT03337802, NCT03419052, NCT02941757, NCT02999152, NCT03625427, NCT01154478); although they are interesting and intriguing, the contribution of OO and/or OOPs in the study was not strictly discernible. For example, study NCT04783714 evaluated the effects of daily consumption of a novel combination of nutraceuticals containing bioactive molecules, in which HTyr was only one of those used in the blend. The clinical trial NCT01381354 used a multimodal therapeutic lifestyle intervention to test its effect on the setting of secondary and primary progressive multiple sclerosis. Once again, HTyr is only one of the components composing the nutritional interventions. Study NCT02941757 evaluated the effect of foods rich in polyphenols in conjunction with brain-training exercises on older adults’ cognitive performance. In this study, nutritional intervention consisted of a diet specifically emphasizing foods high in polyphenols, in which olive oil was only one of the nutritional sources identified (e.g., berries, nuts, cocoa, black beans, and green leafy vegetables). Clinical trial NCT02999152 was excluded as the OOP metabolites, such as 3-sulfate hydroxytyrosol and tyrosol sulfate, have been analyzed as biomarkers in blood and urine samples of patients totally or partially exposed to radiation.

**Figure 3:**
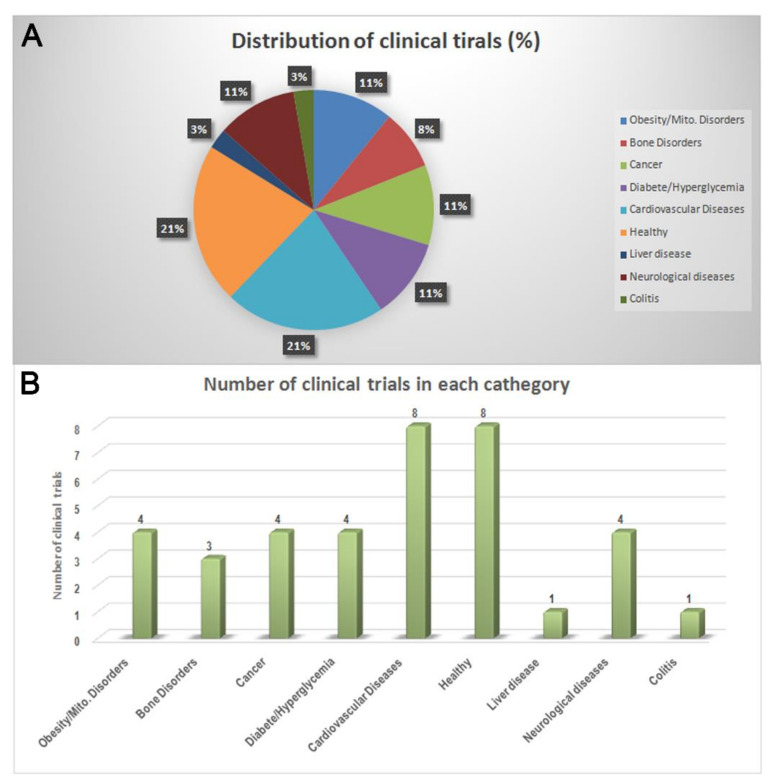
(**A**) The number and (**B**) percentage of OO- and OOP-based clinical trials classified according to the indications provided by clinical trials database. Data from http://www.clinicaltrials.gov/ (15 March 2021).

The remaining 37 studies were considered. We grouped them into nine categories, which were defined according to the indications provided in the item “condition” reported in ClinicalTrials.gov (15 March 2021) (Table 1). This information considers the disease, disorder, syndrome, illness, or injury that is being studied as well as any health-related issues (www.clinicaltrials.gov; 15 March 2021). Accordingly, the most representative categories containing the higher number of studies are “Healthy” and “Cardiovascular Diseases” (*n* = 8 studies; Figure 3A,B).

**Figure 4:**
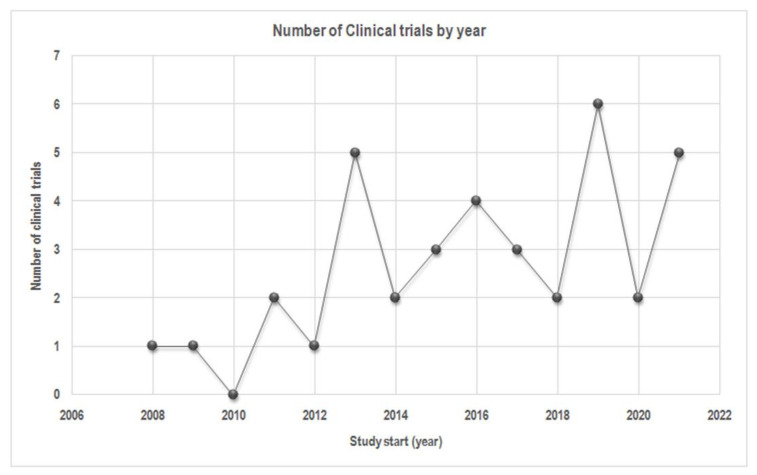
The number of clinical trials classified according to “Study Start.” Data from http://www.clinicaltrials.gov/ (15 March 2021).

We also grouped the 37 studies according the “Study Start” supplied by ClinicalTrials.gov (15 March 2021), which consists of the date on which the first participant was enrolled in a clinical study or the estimated date that the researchers posed as the study start date (Figure 4). This showed that the start dates of 37 clinical trials ranged from 2008 to 2021. As time passed by, the number of studies increased; the maximum number of clinical trials in one year occurred in 2019 (*n* = 6). This is proof of an increasing interest in the role of OOPs on human health.

The identifier numbers of the 37 studies were also used for a Medline search to analyze the available results. Out of them, five studies were found on PubMed as of 15 March 2021.

In 2015, Pérez-Mañá et al. (NCT01788670) provided the results obtained in their study, which focused on the interaction between ethanol and dopamine metabolism for HTyr generation [77]. Their hypothesis was based on previous findings in animals showing an increase in HTyr, a minor metabolite of dopamine, following ethanol intake. To confirm this evidence, they enrolled 24 healthy male volunteers in a double-blind randomized controlled study. Three different cohorts were set up encompassing subjects who received double doses of ethanol or a placebo. Six different doses of ethanol were considered in the study, i.e., 6, 12, 18, 24, 30, and 42 g. The parameters considered by the authors were the urinary excretion of HTyr, Tyr, DOPAC, and homovanillic acid (HVA) as well as the ethanol plasma levels along a 6 h period. Interestingly, the results obtained in the study showed that ethanol administration induced a dose-related increase in urinary excretion of HTyr and Tyr. The authors speculated an endogenous production of these molecules by the shifts in the dopamine and tyramine oxidative metabolism due to the ethanol administration [77].

Study NCT02273622, “Nutritional Intervention Study to Evaluate the Effect of Hydroxytyrosol on Phase II Enzymes in Healthy Subjects,” was set up to analyze the effects of HTyr on Phase II enzyme expression. In particular, Crespo et al. investigated an alternative hypothesis to the notion that polyphenols act as direct free radical scavengers [90]. They aimed to demonstrate that these molecules may be processed as xenobiotics once adsorbed, activating Phase II enzyme expression through the Keap1/Nrf2/Are signaling axis. The study design was a double-blind randomized trial in which volunteers tested two HTyr doses (5 and 25 mg 7 d) vs. a placebo. The analyses carried out on peripheral blood mononuclear cells of the cohort did not reveal modifications in Phase II enzyme expression. This suggests that the hormesis hypothesis should be investigated further in future trials [90].

Filip et al. reported the results obtained in “A Randomised, Double Blind, Parallel Group, 12-month Comparison of a Standardized Olive Extract with Placebo in Postmenopausal Women with Decreased Bone Mineral Density” (Study NCT00789425). The authors focused on osteoporosis, which is a skeletal disorder affecting bone turnover and disturbing its strength [91]. The aim of the study consisted in providing insights about the effect of OOPs on bone turnover in osteopenic postmenopausal women. This hypothesis is based on preclinical studies evidencing the role of OOPs in increasing osteoblast activity. The contribution of Ole in limiting the adipocytic differentiative ability in favor of the osteogenic phenotype was evidenced in mesenchymal stem cells, multipotent adult stem cells giving rise to osteoblast and adipocytes [92]. Drira et al. demonstrated the inhibitory effect of HTyr and Ole on the adipocyte differentiation in 3T3-L1 cells [93]. Thus, a double-blind control-placebo study was performed on a cohort of 64 osteopenic patients with a median age of 59.5 ± 4.9 years. Treatment consisted of a 12-month daily administration of olive leaf extract standardized for Ole content (>40%) and 1000 mg Ca; the control group received only 1000 mg Ca (placebo). After 12 months, the authors described an increase in the levels of osteocalcin, a pro-osteoblastic marker, and a significant decrease in total and LDL cholesterol in the treatment group when compared to the placebo one. Again, a significant change in the bone mineral density of the lumber spine was observed in the placebo group, while this parameter seemed to be stable in the treatment group. Although this is a limited-scale prospective study, these results seemed to confirm the promising biological activities of OOPs in maintaining the balance of bone-turnover [91]. Further studies need to be performed in order to understand the therapeutic potential of OOPs.

In 2020, Mosca et al. published the results of their study, which focused on pediatric non-alcoholic fatty liver disease (NAFLD) [94]. This is a complex disease resulting from a series of liver injuries. Oxidative stress and low-grade systemic inflammations seem to play an important role in disease progression toward non-alcoholic steatohepatitis (NASH). For this reason, many trials in the last decade have investigated the beneficial contribution of antioxidant and inflammatory molecules for NAFLD. In particular, vitamin E (VitE) showed promising improvements in biochemical and histological parameters in NAFLD patients. Nevertheless, the low bioavailability of these molecules seemed to be an important limitation, so the authors in their study proposed a combinatory approach with another phytochemical, HTyr. In particular, they provided the results of observational conclusions obtained by including children with NAFLD in a double-blind placebo-controlled trial consisting of 4-month daily administrations of HTyr and VitE (3.75 mg and 5 mg, respectively; Study NCT02842567). Plasma levels of IL-6, Il-1β, IL-10, TNF-α, 4–hydroxy-2-nonenal (4-HNE), and 8-hydroxy-2′deoxyguanosine (8-OHdG) were analyzed in the cohort of children. Interestingly, four months of HTyr + VitE administration induced an increase in IL-10 level with respect to the placebo group, with a concomitant decrease in 4-HNE and 8-OHdG. These findings let the authors speculate that a combinatory treatment of HTyr and VitE seemed to contribute to reducing NAFLD-related inflammation and oxidative stress [94].

Sanchez-Rodriguez published two papers in 2018 and 2019, which reported the results of the study “New Industrial Procedures for Achieving a Nutritional Added Value of the Olive Oil. The NUTRAOLEUM Study” (NCT 02520739) [95,96]. The study evaluated the health properties of virgin olive oils (VOOs) enriched with phenolic compounds and triterpenes. A cohort of healthy volunteers was daily supplemented for three weeks with 30 mL of three oils: a VOO of 124 ppm phenolic compounds and 86 ppm of triterpenes; an optimized VOO (OVOO) of 490 ppm phenolic compounds and 86 ppm of triterpenes; and a functional OO (FOO) of 487 ppm phenolic compounds and 389 ppm of triterpenes. The trial was conducted as a randomized, cross-over, controlled, double-blind, intervention study.

In 2018, Sanchez-Rodriguez analyzed the effect of the bioactive molecules on biomarkers for metabolic syndrome and endothelial function. The authors showed that VOO enriched in phenolic compounds increased the levels of plasma high-density lipoprotein cholesterol (HDLc) in female subjects. HDLc is one of the features of metabolic syndrome. Again, their results evidenced that VOO, with at least 124 ppm of phenolic compounds regardless of the triterpene content, improved endothelin-1 levels in vivo and ex vivo [96].

In the other paper, the effect of triterpenes (oleanolic and maslinic acids) on decreasing DNA oxidation and plasma inflammatory biomarkers were described. Indeed, the intervention of FOO, rich in both phenolic compounds and triterpenes, seemed to induce a reduction in the levels of urinary 8-OHdG, IL-8, and TNF-α with respect to OVOO [95]. 8-OHdG is a sensitive biomarker for DNA oxidative damage [97] while IL-8 and TNF-α are pro-inflammatory cytokines [98].

Despite the study’s limitations, e.g., the choice of a target population consisting of young and healthy subjects [96], the overall results described by Sanchez-Rodriguez pose intriguing findings about the effect of OO bioactive molecules on human health.

All the evidence reported in the paragraph demonstrate the growing interest about the healthy properties of OOPs. The number of clinical trials based on the principal poly-phenols such as HTyr, Tyr, and Ole has grown rapidly in this decade, and it is proof of the clinical potential of these molecules. Indeed, the studies listed in this section cover a wide range of aspects and diseases in which these molecules are under investigation.

## 6. Conclusions

The amount and distribution of polyphenols in olive and OO varies, depending on some features such as pedoclimatic, agronomic, and technical conditions. Indeed, different OO phenolic profiles have been described [7]. According to this evidence, in-depth studies are needed to provide useful insights to standardize the polyphenolic distribution and understand the variables associated with phenol-enriched OO.

In addition to polyphenol content in OO, other factors influence these variations and deserve to be analyzed to fully understand the healthy potential of OOPs. As discussed above, variations in the bioavailability and tissue distributions of OOPs and differences in gut microbiota could influence the biological activities of these compounds and/or the potential beneficial effect of OO. Moreover, plasma and tissue concentrations of OOP metabolites often reach higher levels than parent compounds [8].

On the whole, these complexities are the issues that need to be challenged in the near future to fully understand the real potential of OOPs and to bring out the beneficial effects of OOs. Further investigations would provide insights about the different profiles of phenolic metabolites and their biological effectiveness along with the possible interactions between these molecules and other nutrients. These findings will be pivotal to set up tailored clinical studies that may be informative concerning the impact of OOPs and their metabolites on human health. An in-depth knowledge of the steps that occur following polyphenol ingestion may reveal useful insights to be used in clinical settings for the prevention and treatment of many diseases. Indeed, many limitations still remain in existing studies, suggesting the need of further clinical trials to overcome these unaddressed areas.

## Figures and Tables

**Table 1:** Main characteristics of the 37 studies considered in this review.

Category	ID	Title	Condition	Study Start
Obesity/Mito. disorders	NCT04149288	Olive Oil Polyphenols and Cardiovascular Health Biomarkers	Healthy Normal Weight Overweight Obese	May 2021
	NCT03101436	Extra Virgin Olive Oil, Red Wine Polyphenols and Fecal Microbiota	Obesity	2 February 2015
	NCT04317079	Effects of Hydroxytyrosol Administration in Anthropometric Parameters in Overweight and Obese Women	Body Weight Visceral Obesity	30 October 2017
	NCT04543968	Clinical Study of Extra-virgin Olive Oil in Mitochondrial Diseases	Mitochondrial Diseases	1 January 2021
Bone disorders	NCT01828944	Olive Oil Polyphenols, Vitamin D, Docosahexaenoic Acid (DHA) and Locomotor Function (PolivD3)	Osteopenia Sarcopenia	December 2012
	NCT03072108	Dietary Supplement for Joint: the OLE Study	Knee Discomfort Knee Pain	24 June 2016
	NCT00789425	Investigating the Effect of Standardized Olive Extract on Bone Turnover Markers in Postmenopausal Women	Osteoporosis Osteopenia	September 2008
Cancer	NCT04027088	Effect of Preoperative Immunonutrition in Upper Digestive Tract	Immunonutrition Gastric Cancer Esophageal Cancer Pancreas Cancer Surgery Complications	10 August 2019
	NCT02520739	New Industrial Procedures for Achieving a Nutritional Added Value of the Olive Oil. The NUTRAOLEUM Study	Cardiovascular Diseases	February 2014
	NCT04215367	Dietary Intervention with High Phenolic EVOO in CLL	Chronic Lymphocytic Leukemia (CLL)	15 December 2018
	NCT02068092	Olive Oil for High Risk Breast Cancer Prevention in Women	Breast Cancer	December 2013
Diabetes/Hyperglycemia	NCT04764786	Polyphenol Enriched Extra- Virgin Olive Oil and Postprandial Glycemia in Type 1 Diabetes (DOP)	Type 1 Diabetes	1 April 2019
	NCT02669693	Effect of Olives on Glycaemic Response in Vivo	Diabetes	December 2015
	NCT03093753	Effect of a Beverage Comprised of Compounds from Olives on Post- prandial Blood Glucose Responses in Healthy Volunteers	Hyperglycemia	July 2016
	NCT04419948	Oleocanthal Rich Olive Oil Acute Effects on Hyperglycemia and Platelet Activation in T2DM	Diabetes Mellitus, Adult-Onset Platelet Dysfunction Postprandial Hyperglycemia Lipidemia Inflammation Oxidative Stress	16 May 2019
Cardiovascular diseases	NCT04760093	A Multicenter Pilot Study to Evaluate the Effect of EVOO on Lipid Parameters	Cardiovascular Diseases	1 March 2021
	NCT01796561	The Effect of Olive Leaf Extract on Blood Pressure in Overweight Prehypertensives	Hypertension	February 2013
	NCT01983943	Olive Oil and Cardiovascular Health	Cardiovascular Disease Endothelial Function	August 2013
	NCT02783989	Effects on Cardiovascular Risk Factors of the Endogenous Hydroxytyrosol Generation After the Combined Intake of Wine and Tyrosol in Humans	Cardiovascular Disease	20 January 2016
	NCT04520126	Effect of Olivomed (Olive Extract) on Endothelial, Cardiac and Vascular Function	Coronary Artery Disease	1 December 2020
	NCT02421835	Olive Leaf Extract as Part of a Healthy Lifestyle in the Reduction of Blood Pressure	Pre-Hypertension	April 2013
	NCT03528603	Acute Assessment of Platelet Reactivity After the Intake of Oleocanthal	Platelet Aggregation Nutritional and Metabolic Disease Cardiovascular Diseases	2 April 2018
	NCT02902913	Impact of Extra Virgin Olive Oil Oleocanthal Content on Platelet Reactivity	Cardiovascular Diseases	January 2015
Healthy	NCT01347515	Bioactivity of Olive Oils Enriched with Their Own Phenolic Compounds (VOHF1)	Polyphenol Absorption in Healthy People	April 2011
	NCT03886597	Nutritional Intervention with Table Olives in Healthy Volunteers	Healthy Biological Availability Nutritional Intervention Functional Food Nutrition Physiology	25 March 2019
	NCT02273622	Human Study of Hydroxytyrosol on Phase II Enzymes in Healthy Subjects	Healthy	October 2014
	NCT01790672	Contribution of Wine Components on Hydroxytyrosol Body Concentrations and Biological Effects	Contribution of Wine Components in Hydroxytyrosol Formation	May 2011
	NCT02042742	Punicalagin and Hydroxytyrosol Mixture on Different Inflammatory Markers	Healthy	April 2013
	NCT01788670	Relevance of the Ethanol Dose in the Generation of Endogenous Hydroxytyrosol	Contribution of Ethanol on Hydroxytyrosol Formation	May 2009
	NCT04328571	Effects of Enzymatic Digestion and Probiotic on Oleuropein Bioavailability	Healthy Subjects	10 February 2020
	NCT04725955	Postprandial Responses to Hydroxytyrosol-enriched Bread	Postprandial Responses	31 January 2021
Colitis	NCT03408847	Monocultivar Coratina Extra Virgin Olive Oil in UC Patients	Ulcerative Colitis Chronic Mild	20 November 2017
Liver disease	NCT02842567	Hydroxytyrosol and Vitamin E in Pediatric NASH	NAFLD	1 April 2017
Neurological diseases	NCT04440020	Management of Dementia with Olive Oil Leaves-GOLDEN	Prevention	5 January 2019
	NCT03362996	Management of Mild Cognitive Impairment Patients with Extra Virgin Olive Oil-MICOIL	Mild Cognitive Impairment	9 November 2016
	NCT03824197	Auburn University Research on Olive Oil for Alzheimer’s Disease (AU-ROOAD)	Alzheimer Disease Cerebral Amyloid Angiopathy	7 May 2019
	NCT04787497	The Effect of Extra Virgin Olive Oil in People with Multiple Sclerosis	Multiple Sclerosis	December 2021

Note: Information was collected at http://www.clinicaltrials.gov/ (15 March 2021).

## Data Availability

Not applicable.
